# The Cytokine Profile in Different Stages of Schistosomiasis Japonica

**DOI:** 10.3390/pathogens12101201

**Published:** 2023-09-27

**Authors:** Xi Wang, Qi Tang, Robert Bergquist, Xiaorong Zhou, Zhiqiang Qin

**Affiliations:** 1 National Institute of Parasitic Diseases, Chinese Center for Disease Control and Prevention (Chinese Center for Tropical Diseases Research), NHC Key Laboratory of Parasite and Vector Biology, WHO Collaborating Centre for Tropical Diseases, National Center for International Research on Tropical Diseases, Shanghai 200025, China; wangxii99@163.com (X.W.); m15970005361@163.com (Q.T.); 2 Ingerod, Brastad, 454 94 Lysekil, Sweden; robert.bergquist@yahoo.se; 3 Hubei Provincial Center for Disease Control and Prevention, Wuhan 430079, China; zxrmed@sina.com

**Keywords:** schistosomiasis japonica, cytokine, interleukin, helper T cells

## Abstract

To explore and profile the level of cytokines in the sera of patients infected with *Schistosoma japonicum* to explore the helper T-cell response of patients either at the chronic or advanced stage of the disease. We randomly selected 58 subjects from several areas endemic for schistosomiasis japonica in China and collected serum samples to be tested for 18 different cytokines secreted by (1) Th1/Th2 cells (GM-CSF, IFN-γ, IL-1β, IL-2, IL-4, IL-5, IL-6, IL-12p70, IL-10, IL-13, IL-18 and TNF-α) and (2) Th9/Th17/Th22/Treg cells (IL-9, IL-17A, IL-21, IL-22, IL-23 and IL-27). The Th1/Th2 cytokines in chronic patients were not significantly different from those in healthy people, while patients with advanced schistosomiasis had higher levels of IL-2, IL-23 and IL-27 and lower levels of IL-18 and IFN-γ. With respect to the Th9/Th17/Th22/Treg cell cytokines, there were higher levels of IL-23. Thus, a limited variation of the cytokine response between the three patient groups was evident, but only in those with advanced infection, while there was no difference between chronic schistosomiasis infection and healthy subjects in this respect. The cytokine expression should be followed in patients with advanced schistosomiasis who show a cytokine pattern of a weakened Th1 cell response and an increased Th17 response.

## 1. Introduction

Schistosomiasis is an important and neglected tropical disease (NTD) affecting about 250 million people in 78 countries [1]. It is a parasitic disease caused by three main species of trematode worms, *Schistosoma mansoni*, *S. haematobium* and *S. japonicum*, that seriously harms human health and affects social and economic development [2,3]. *S. mansoni* is found in Latin America, *S. mansoni* and *S. haematobium* in Africa, while *S. japonicum* is distributed in China and the Philippines, with a minor incidence in Indonesia [4,5]. The parasite uses freshwater snails as intermediate hosts, and human infection occurs through water contact during professional or leisure activities. After infection, female and male worms develop in the human, the definite host, where they pair up and produce thousands of eggs per day to be released via feces, closing the life cycle by infecting the snails that also act as a parasite reservoir [3].

With the rise in ecotourism and travel to remote areas, the risk of exposure to NTDs, including schistosomiasis, has increased. Schistosome cercariae, released from the intermediate snail host, penetrate the skin of the mammalian host and migrate towards the intestines while maturing into microscopic worms. Soon after the cercarial penetration, infected people often experience a temporary, symptomatic, acute stage called Katayama fever [3]. The adult worm is fairly well camouflaged as it covers its outside with proteins from the definite host, so the pathology caused is mainly the result of immune responses against schistosome eggs not excreted from the human body but trapped in the tissues, primarily the liver [2,3]. The typical granulomatous reactions mark the chronic stage of the disease, which without treatment proceeds to the advanced stage as the granulomas are gradually replaced by fibrous tissue. Ultrasonography reveals typical streaks in the lever parenchyma that eventually result in portal-vein dilatation, which is in most cases irreversible. The severity of the symptoms is related both to intensity of infection and to individual immune responses [6,7].

The responses against the eggs are prodded by various signaling compounds (cytokines), but when the infections are not swiftly overcome, this complicated system can overreact and lead to severe damage [8]. A brief description of how the system operates starts with the presentation of processed antigens to naive CD4+ T cells, which become activated and differentiate into distinct T-helper cell (Th) subsets termed Th1 and Th2, etc. Interleukin-12 (IL-12) and interferon-gamma (IFN-γ) promote the differentiation of activated CD4+ T cells into Th1 cells [9,10], while interleukin-4 (IL-4) promotes their differentiation into Th2 cells [11]. In the early stage of infection, this immune response is modulated towards a Th1-mediated immune mechanism by interferon-gamma (IFN-γ), but when an infection establishes itself in the host, the overall response gradually develops towards a Th1/Th2 balance [9]. However, a full description of the cytokine system is much more complicated than that, with the cytokines released from the Th2 subset also capable of causing tissue damage in allergy and asthma as well as specific action against helminth infections [11].

How this system operates against schistosome infection has largely been illuminated in experimental animal systems [9,12,13,14,15,16,17,18], but an increasing number of studies now also deal directly with the human immune responses elicited by schistosome infection. For example, it has been shown that Th17 cells [19], regulatory T-cells (Tregs) [20], and follicular helper T-cells (Tfhs) [21] also participate in immune regulation in human schistosomiasis, with cytokines secreted by these cells promoting the inflammatory pathological responses induced by the schistosome eggs. Thus, schistosomiasis patients at different stages of the infection express different cytokine spectra. During the acute phase of the infection, a Th1 response characterized by increased levels of IFN-γ, tumor necrosis factor-alpha (TNF-α), interleukin-1 (IL-1) and IL-6 is elicited, later modulated towards the Th2 side of reactions, i.e., production of immunoglobulin E and eosinophilia driven by IL-4 and IL-5 [22,23].

Cytokines may be detected by the enzyme-linked immunosorbent assay (ELISA) or by radioimmunoassay (RIA) [24,25]. In addition, liquid chip technology has been widely used for rapid biomarker quantification. The significant advantages of this approach are high sensitivity, high throughput, and small a sample size required, allowing the quantification of up to 100 unique target compounds in a single well of a test plate [26,27]. In this study, liquid chip technology was used to detect the expression profile of 18 cytokines in the sera of patients with schistosomiasis japonica in order to explore the cytokine expression spectrum in patients, thereby elucidating the characteristics of the immune response to schistosome infection at different stages of the disease.

## 2. Materials and Methods

### 2.1. Study Design and Participants

This study was conducted on patients from the major endemic areas of schistosomiasis japonica in China, including Hubei, Hunan and Jiangxi Provinces. The study included 28 patients with chronic schistosomiasis from Hubei and Hunan provinces and 12 patients with advanced schistosomiasis (also called late-stage schistosomiasis) from the Duchang Schistosomiasis Control Hospital in Jiangxi Province. All schistosomiasis patients were tested for *S. japonicum* eggs in their stools using the Kato–Katz method [28], immunological diagnosis [29] and a B-ultrasound examination to assess the severity of the disease. Eighteen people from the Affiliated Hospital of Yiyang Medical College in Hunan Province were added as healthy controls since they had no signs or symptoms consistent with schistosomiasis, no history of contact with infectious water, and their fecal Kato–Katz and serological tests were both negative.

### 2.2. Sample Processing

Between 2 and 4 mL of whole blood without anticoagulant was collected from each subject and left for 20–30 min at room temperature (20–25 °C). The sera were collected after centrifugation at 1000× *g* for 10 min at 4 °C (hyperlipidemic samples were centrifuged at 10,000× *g* for 10 min) and then immediately transported to the laboratory of the National Institute for Parasitic Infections (NIPD) at the China Center for Disease Control and Prevention (China CDC) in Shanghai by cold chain for follow-up detection and analysis. To avoid repeated freezing–thawing, if they could not be processed within 24 h, the samples were stored below −20 °C.

### 2.3. Cytokine Detection

Sera were analyzed for 18 cytokines using human Th1/Th2/Th9/Th17/Th22/Treg 18-Plex HumanProcartaPlex™ (EPX180-12165-901) with sets of antibodies and standards from Thermo Fisher (Waltham, MA, USA). The aim was to explore the helper T-cell response in patients at the chronic and advanced stage of the disease. The target list was (1) with reference to secretions from Th1/Th2 cells (the granulocyte macrophage colony-stimulating factor (GM-CSF), IFN-γ, TNF-α, IL-1β, IL-2, IL-4, IL-5, IL-6, IL-10, IL-12p70 (a heterodimer composed of p40 and p35 subunits), IL-13 and IL-18) and (2) with reference to secretions from Th9/Th17/Th22/Treg cells (IL-17A, IL-21, IL-22, IL-23 and IL-27). The biological significance and medical relevance of these cytokines are given in the Supplementary Materials. The ProcartaPlex panel enables exploration of the T-helper response by analyzing 18 targets in a single well using xMAP technology from Luminex (Austin, TX, USA) according to Ashby et al. [30].

**Figure 1:**
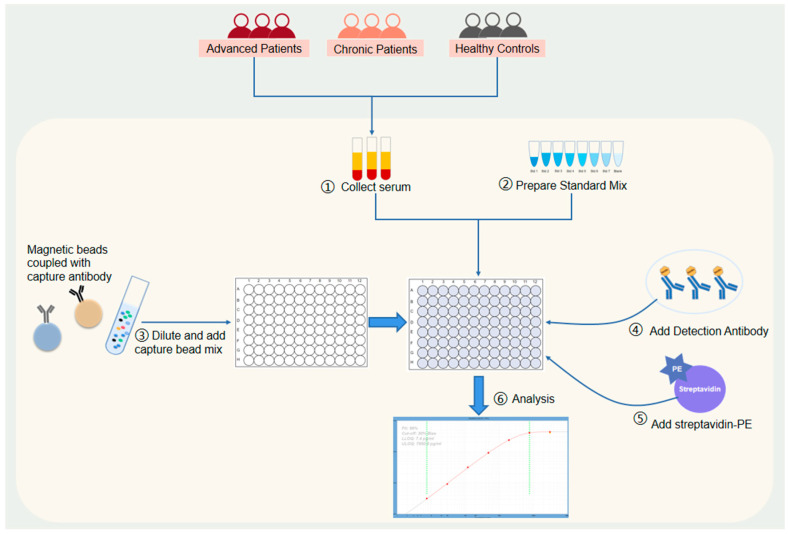
The cytokine detection procedure.

The procedure for detecting cytokines using magnetic beads technology from Luminex^TM^ is shown in Figure 1. Briefly, an antigen standard was first prepared and diluted. Each vial of standard was reconstituted with 50 µL of sample-type-specific buffer. A 4-fold serial dilution of the reconstituted standards were prepared using the PCR 8-tube strip provided. Then different kinds of magnetic beads coupled with capture antibodies were mixed and added into the 96-well microplates. The plate was then washed using a hand-held magnetic plate washer and wash buffer. Next, an amount of 25 µL of Universal Assay Buffer (1×) was added to each well, followed by 25 µL of prepared standards or samples into dedicated wells. For wells designated as blanks, an additional 25 µL of Universal Assay Buffer was added in place of the serum or plasma samples, after which the plate was sealed with a plate seal then covered with a black microplate lid and shaken at 500 rpm for 60–120 min at room temperature. The plate seal was remove and discarded after incubation and the plate was washed twice. Next, 25 µL of the detection antibody mixture (1×) was added to each well and mixed with the beads provided, followed by resealing, applying the black microplate lid and incubation for 30 min on a plate shaker (500 rpm) at room temperature. Then 50 µL of streptavidin-PE (SAPE) was added and the plates were sealed again, incubated at room temperature and underwent gentle movement for 30 min. Finally, 120 µL of reading buffer was added into each well, after which the plates were resealed and gently moved for 5 min at room temperature and then read on the xMAP™ instrument. Figure 1 shows the procedure followed.

### 2.4. Ethics Approval

Informed consent was obtained from each study participant, and the collection and use of serum samples for this study were approved by the Human Ethics Committee of NIPD, Chinese CDC, Shanghai, China.

### 2.5. Data Analysis

Descriptive analyses, sample frequencies and the distribution of results were evaluated. Statistical analysis was performed using SPSS software, version 17. Non-parametric tests were used for data with non-normal distributions and heterogeneity of variance. Kruskal–Wallis H tests were used for the comparison of multiple groups. Chi-square tests was used for the analysis of qualitative data, adopting a 5% significance level (*p* < 0.05).

## 3. Results

### 3.1. Patient Characteristics

The age and sex distribution of the patients is shown in Table 1. The mean ages of the chronic schistosomiasis patients, late-stage patients and healthy controls were 54.8 ± 1.65, 49.7 ± 1.26 and 47.6 ± 2.96 years, respectively. There were no statistically significant differences with respect to gender and age among the three groups (*p* = 0.631; *p* = 0.113), suggesting that the baseline data were consistent and comparable.

### 3.2. Cytokines Secreted by Th1 cells

**Figure 2:**
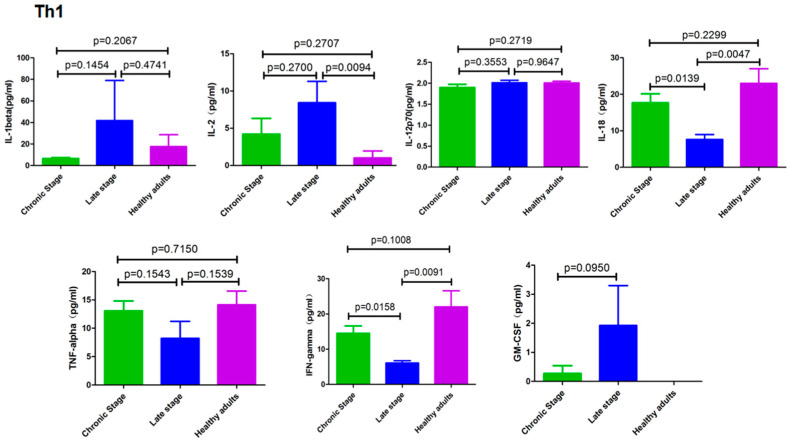
Cytokines secreted by Th1 cells from healthy controls and patients with different stages of schistosomiasis japonica.

As seen in Figure 2, there was no difference in the expression levels of the Th1 cytokines between chronic schistosomiasis patients and healthy subjects, but patients with advanced schistosomiasis had higher levels of IL-2 than those in the healthy group. In addition, patients with advanced schistosomiasis had lower IL-18 and IFN-γ levels than those with chronic schistosomiasis and the healthy controls. The remaining four cytokines (IL-1β, IL-12, GM-CSF and TNF-α) showed no significant difference among patients in the different clinical stages and the controls.

### 3.3. Cytokines Secreted by Th2 Cells

**Figure 3:**
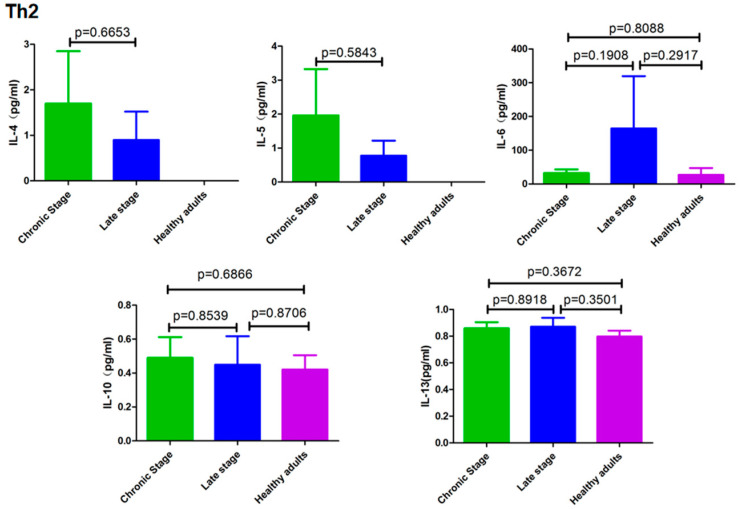
Cytokines secreted by Th2 cells from healthy controls and patients with different stages of schistosomiasis japonica.

Figure 3 shows that the levels of cytokines secreted by Th2 cells. IL-4, IL-5, IL-6, IL-10 and IL-13 did not differ significantly among the three groups under study (*p* > 0.05).

### 3.4. Cytokines Secreted by Th9/Th17/Th22/Treg Cells

**Figure 4:**
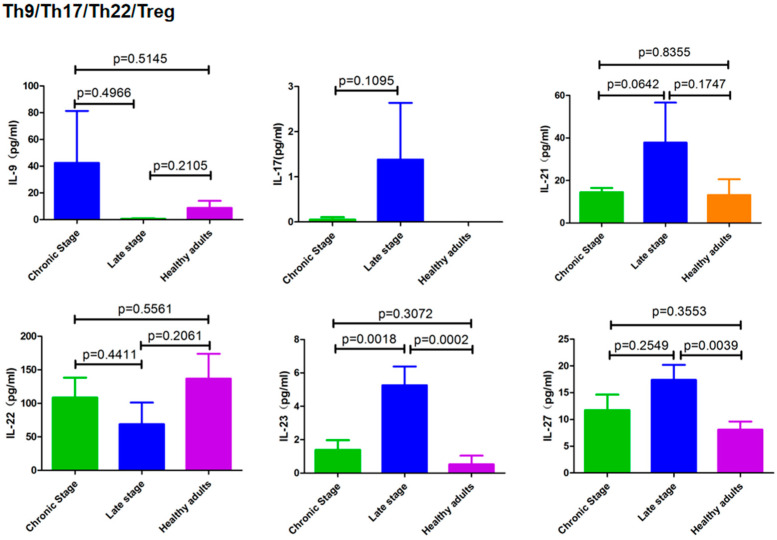
Cytokines secreted by Th9/Th17/Th22/Treg cells in healthy controls and patients with different stages of schistosomiasis japonica.

High levels of IL-23 were found in patients with advanced schistosomiasis, while IL-17 was only moderately elevated in these patients and not at a statistically significant level. IL-27 showed an increasing trend, with a significant difference between the patients with advanced schistosomiasis and the healthy controls, but no statistical difference was found compared with the chronic patients (Figure 4).

## 4. Discussion

The fine tuning of the immune response depends on the context of the infection and the ability of the immune system to appropriately regulate its responses. Indeed, the balance between Th1 and Th2 responses is essential for overall immune health, and imbalances have been shown to potentially result in various immune-related diseases, including autoimmunity (excessive Th1 response) [31,32,33,34] and allergies (excessive Th2 response) [11].

The pathology of schistosomiasis is initiated by the deposition of schistosome eggs in the liver, intestine and other tissues of the host. Under the continuous stimulation of egg antigens, inflammatory and immune cells are sequentially recruited to the infected site, leading to the formation of granulomas around the eggs and eventually fibrosis [35]. This process is closely related to the response of the cytokine network. Various cytokines promote or suppress specific immune responses and play an important regulatory role in the process of tissue fibrosis [36]. The production of central vein fibrosis or peripheral portal vein fibrosis, which are key signs of both chronic and advanced schistosomiasis, depends on different key cytokines [37].

It seems that the Th1 immune response is weakened in the advanced stage. For example, experimental studies on *S. mansoni* have shown that schistosome infection in the absence of IL-4 leads to severe, fatal disease as opposed to the chronic form, which is typified by the neutralization of IFN-γ [38], a finding later supported by a clinical study [39]. In line with these results, we found that the level of serum IFN-γ was lower in patients with advanced schistosomiasis japonica compared to patients with only mild fibrosis. IL-18 induces IFN-γ together with IL-12 or IL-15 and plays a role similar to IFN-γ [40,41,42], which is supported by our results showing that the expression of IFN-γ and IL-18 in late-staged schistosomiasis patients was significantly lower than that in chronic patients and healthy people.

IFN-γ inhibits the differentiation of Th17 cells [43]; therefore, the decreased IFN-γ in the sera of patients with advanced schistosomiasis may lead to Th17 differentiation. In recent years, the role of Th17 cells in advanced schistosomiasis has attracted increasing attention, as Th-17 has been shown to play an important pathogenic role in inflammation and infection [14]. This cytokine is directly related to the severity of granulomatous inflammation, suggesting that it is one of the pathogenic factors of disease development in intestinal schistosomiasis. Thus, IL-17-producing T cells driven by IL-23 may be the main force leading to the pathology seen in the liver involvement of chronic and advanced schistosomiasis [12,44]. IL-23 and IL-1β are key factors involved in the generation of antigen-specific Th17 cells in the pathogenesis of severe schistosomiasis [18]. When anti-IL-17 neutralizing antibodies were administered to mice immunized with SEA using complete Freund’s adjuvant, a significant reduction in the severity of immunopathological reactions was observed [44]. Similarly, Zhang et al. [43] found that neutralizing IL-17 with anti-IL-17 monoclonal antibodies significantly alleviates liver immunopathology and hepatocyte necrosis induced by *S. japonicum* eggs.

The fact that IL-17 and IL-23 cytokines are also involved in the development of autoimmune diseases [12,31,32] is of particular interest as we, in a previous study [34], found positive rates of the antinuclear antibody (ANA) in 70% of patients with advanced schistosomiasis. Additionally, a variety of other autoantibodies against different autoantigens were also detected in these patients (data unpublished). These findings suggest that the IL-23/Th17 axis and/or the IL-27 pathway may play an important role in the immune pathogenesis and autoimmunity of patients with advanced schistosomiasis. In this study, serum IL-2 was elevated in patients with advanced schistosomiasis compared with the healthy controls, which seems to be significant in maintaining the number and function of Treg cells and inhibiting autoimmunity. The association between schistosomiasis and autoimmunity is complex, and the current study represents only the first step in a larger approach focusing on the expression and function of multiple cytokines and their combinations with respect to the simultaneous presence of autoimmune disease and schistosomiasis.

In summary, we found significantly lower levels of IL-18 and IFN-γ and higher levels of IL-23 in patients with advanced schistosomiasis compared with healthy controls and chronic patients. However, it is worth noting that our cytokine profile findings seem to be different from those seen in schistosomiasis mansoni, which has shown higher levels of IL-9, IL-10 and IL-17 in patients with advanced liver fibrosis [15]. However, we did not find that IL-9 and IL-10 increased in patients with advanced schistosomiasis japonica, which may be due to the differences in the cytokine response between the two species. Previous studies have also indicated differences in the underlying pathological mechanisms of the different species, e.g., when IL-4-deficient mice were infected with *S. japonicum*, they survived longer than normal mice [17], while IL-4-deficient mice infected with *S. mansoni* had a higher mortality rate [38].

Most of these signaling substances emanate from CD4+ T cells, which play a key role in the establishment of the cytokine environment and are essential for the immune response during helminth infections [12]. Experimental animal studies have shown that a specific group cytokine subset (IL-4, IL-13, IL-17 and IL-23) is stimulated by the presence of schistosome eggs [14,17] and that the IL-23/IL-1/IL-17 axis plays a central role in the immunopathology in this situation [18]. IL-23 cannot on its own promote the development of IFN-γ but is an important cytokine upstream of IL-17 and is thus one of the necessary factors for the expansion of pathogenic CD4+ T cells that produce IL-17, IL-6 and TNF-α [45,46]. As a large number of *S. japonicum* eggs end up in the liver, this organ becomes fibrotic due to the many cytokine-induced granulomas around them. High IFN-γ levels are associated with a significant reduction in the risk of fibrosis, while high levels of TNF-α are associated with an increased risk of periportal fibrosis [39]. The modulation of IFN-γ production and signaling pathways could therefore provide new targets for the treatment and control of schistosomiasis.

The were several limitations that could not be avoided at this stage of research. For example, a larger number of patients would have made it possible to produce a proper case-control study considering both age and gender and also made it possible to rule out the potential of immune senescence as a confounder. At this stage of our research, it was felt sufficient to measure the cytokine serum levels to provide indications for follow-up testing with cell cultures, as this will produce more specific cytokine responses. Another aspect is the inadequacy of current diagnostics. Schistosomiasis is diagnosed by finding eggs in the stools (urine in the urogenital form of the infection). However, false-negative results cannot be avoided due to the comparatively low sensitivity of the microscope. In addition, serological tests cannot distinguish between current infection and previous exposure, and many remain serologically positive for several years after treatment [3,7]. Diagnostic inaccuracy could have misled us but only in a limited number of cases.

## 5. Conclusions

This study revealed significant changes in the expression levels of some Th1- and Th17-type cytokines in advanced schistosomiasis japonica, indicating that there is a set correlation between the immuno-pathological responses to *S. japonicum* infection and the way the cytokine network operates during this specific infection.

## Figures and Tables

**Table 1:** Characteristics of schistosomiasis patients and controls.

Patient/Disease Group	Age(Mean ± s)	Gender	
		Male	Female
Chronic stage	54.8 ± 1.65	18 (64.3%)	10 (35.7%)
Late stage	49.7 ± 1.26	7 (58.3%)	5 (41.7%)
Healthy adults	47.6 ± 2.96	9 (50%)	9 (50%)

## Data Availability

The data are available from the corresponding author on reasonable request.

## Supplementary Materials

The following supporting information can be downloaded at: https://www.mdpi.com/article/10.3390/pathogens12101201/s1, Table S1: Biological effects of 18 kinds of Cytokines. References [15,18,32,33,41,42,47,48,49,50,51,52,53,54,55,56,57,58,59,60,61,62,63,64,65,66,67,68,69,70,71,72,73,74,75,76,77,78,79,80,81,82] are cited in the supplementary materials.
