# Supplementary material for: The Cytokine Profile in Different Stages of Schistosomiasis Japonica
Source: Pathogens. 2023 Sep 27;12(10):1201. doi: 10.3390/pathogens12101201 (PMC10610117; doi:10.3390/pathogens12101201)
Supplement: Supplementary file 1 [file pathogens-12-01201-s001.zip › pathogens-2537043-supplementary.pdf]

Table S1: Biological effects of 18 kinds of Cytokines.

Cytokines	Biological effects
GM-CSF	GM-CSF is a hematopoietic growth factor. There are many factors that affect GM-CSF production, for example, IL-1β, IL-23, IL-12, TNF-αand TLR promote its secretion, while IFN-γ, IL-4 and IL-10 inhibit its production. GM-CSF was also found to play an important role in pro-inflammatory effects and it is essential for the pathogenicity of CD4 T cells [47]. El-Behi et al. demonstrated that IL-23 induced GM-CSF production in Th17 cells and these produced GM-CSF contributed to the maintenance of Th17 cells phenotype and their functional maturation. In addition, data suggested that GM-CSF induced IL-23 in APC, then IL-23 induced Th17 cells to express GM-CSF, which in turn stimulated IL-23 production in APC, forming a positive feedback regulatory pathway that lead to an amplification of the inflammatory response [48]. The pathogenic role of GM-CSF is reflected in autoimmune diseases that depend on cellular immune responses. GM-CSF-expressing B cells may contribute to autoantibody production and pathogenesis of rheumatoid arthritis [49].
TNF-α	TNF-α was initially found to be an anti-cancer agent, but it is also a pleiotropic cytokine that can produce different effects under various physiological or pathological conditions. TNF-α contributes importantly to the development of T cells, B cells, and dendritic cells. However, TNF-α is also a potent inflammatory mediator and apoptosis inducer. TNF-α and its family members have been implicated in some diseases, including cancer, neurological diseases, cardiovascular diseases, lung diseases, autoimmune diseases and metabolic diseases. TNF-α may promote immune regulation disorders and may be a potential factor leading to autoantibody induction[50-51].
IFN-γ	The production of IFN-γ is promoted by IL-12 and IL-18, while its production is inhibited by IL-4, IL-10, TGF-β, etc. [52]. The production of IFN-γ is the characteristic of the Th1 response. IFN-γ has an important role in host defense and can activate neutrophils and macrophages to eliminate pathogens [42]. It was found that in schistosoma-infected mice, some IFN-γ and IL-4-scrected cells were present in hepatic granulomatous inflammation and around schistosome eggs, indicating that schistosome infection can induce large amounts of IFN-γ and IL-4 in mouse liver T lymphocytes [53]. In addition, IFN-γ is also associated with SLE. Increased IFN-γ in SLE patients results in a phenotype biased towards Th1 and Th17 in the CD4+ T cell population, which plays an important role in the pathogenesis of SLE [54].
IL-1β	IL-1β plays a critical and pleiotropic role in coordinating innate and adaptive immune responses. It is involved in pathogen clearance and immune homeostasis. However, aberrant IL-1β production is associated with monogenetic auto-inflammatory diseases, but also with ligand-dependent, multifactorial, sterile inflammatory conditions, such as atherosclerosis, type 2 diabetes, Alzheimer's disease, or gout[55]. IL-1β is mainly produced by innate immune system cells such as monocytes and macrophages. It is first produced as an inactive 31 kDa precursor called pro-IL-1β, which becomes active only after cleavage by caspase-1 and release from the cell [56]. The T2 ribonuclease omega-1 secreted by mansoni Schistosoma eggs is a powerful

	Th2-inducing factor that enhances the secretion of IL-βin macrophages stimulated with TLR, and this process dependends on the activation of inflammasome such as NLRP3/ASC/caspase-1 inflammasome [57]. IL-1β can significantly upregulate GM-CSF production in Th1 and Th17 cells [48]; and can also modulate IFN-γ-mediated responses and efficiently induce COX-2, leading to a large amount of PGE2, which in turn inhibits IFN-γ production. IL-1β promotes polarization towards a Th17 response by inhibiting IFN-γ and Th1 immunity [41].
IL-2	The basic function of IL-2 is to control the immune response and maintain self-tolerance. IL-2 is essential for the maintenance of Foxp3 regulatory T cells (Treg cells). When IL-2 or its related receptor signaling is lacking, the number and function of Treg cells are impaired, which is one of the reasons for human autoimmunity [58]. Additionally, IL-2 can inhibit Th17 cells and follicular helper T cells. It is reported that low-dose IL-2 has the potential to treat autoimmune diseases [59]. IL-2 inhibits GC formation and autoantibody production by limiting differentiation of follicular helper T cells. Therefore, low-dose IL-2 treatment aims to compensate for the lack of IL-2 or to enhance the Treg population. However, high-dose IL-2 therapy may lead to severe toxicity and side effects [59].
IL-4; IL-13	IL-4 and IL-13 play a key role in allergic inflammation and parasitic infection. These cytokines have the ability to switch IgE and IgG4 immunoglobulins. They stimulate B cell proliferation, as well as the activation of eosinophils, basophils and mast cells. In addition, they are involved in the production of collagen by fibroblasts and induce the expression of vascular cell adhesion molecule (VCAM) -1 on endothelial cells[60]. Usually, Th2 and IL-4 are the main initiators of schistosomiasis-induced liver fibrosis. The experimental results showed that IL-4 rised rapidly after four weeks of schistosomiasis infection and peaked at 12 weeks [61]. IL-4 and IL-13 are critical in early immune responses against mansoni Schistosoma eggs and in preventing lethal infection. Genetic deletion of IL-4 was found to lead to a Th1-prone immune response and severe hepatotoxicity, even rapid death, which was more pronounced in IL-4/IL-13-deficient mice [62].
IL-5	IL-5 is mainly produced by Th2 cells. It has the function of inducing the differentiation of B cells into antibody-secreting cells, as well as an enhancer of the differentiation and proliferation of eosinophilic progenitor cells. High level of eosinophilia that IL-5-induced plays an important role in many allergic diseases, so targeting IL-5 signaling would be a potential therapeutic modality [63-64]. When infected with schistosomiasis, the mice liver T cells can induce large amounts of IL-5. Meanwhile, about one-third of IL-5(+) Th cells can express IL-4, and 10% can produce IFN-γ or IL-17A. IL-5-producing Th cells possessed some properties that differed from other cytokine-secreting Th cells [65].
IL-6	IL-6 is considered to be an important factor in promoting the differentiation of B cells into plasma cells.During antigen stimulation, IL-6 is a very specific and potent inducer of IL-21 production by CD4 T cells. It indirectly promotes antibody production in B cells by upregulating IL-21 expression [66]. The rapid production of IL-6 contributes to host defense during infection and tissue damage, but the excessive synthesis of IL-6 and the imbalance of IL-6 signaling pathway are related to disease

	pathology. IL-6 blockade strategy is beneficial for several inflammatory diseases, such as RA, Castleman`s disease, juvenile idiopathic arthritis(JIA), and cytokine release syndrome(CRS) induced by chimeric antigen receptor T cell therapy[67]. IL-6 cooperates with TGF-β can promote the differentiation of naive CD4 T cells into Th17 cells, but at the same time IL-6 also inhibits TGF-β-induced Treg differentiation. Upregulation of this Th17/Treg balance disrupts immune tolerance, leading to the development of autoimmune and chronic inflammatory diseases [68].
IL-10	IL-10 is an important anti-inflammatory cytokine that plays a central role in the regulation and termination of inflammatory responses[69]. It also has a direct inhibitory effect on memory Th17 and Th2 cells, while promoting the survival and function of Tregs. Impaired IL-10 signaling pathways are associated with inflammatory diseases such as inflammatory bowel disease (IBD) and is often accompanied by immunopathology during infections; conversely, high or dysregulated production of IL-10 may lead to chronic infection[70].
IL-12	IL-12 is a pro-inflammatory cytokine produced by dendritic cells, macrophages and B cells in response to microbial pathogens [71]. This cytokine regulates T cell and NK cell responses, induces IFN-γ production, favors helper Th1 cells and is an important link between innate resistance and adaptive immunity [72]
IL-18	IL-18 is a kind of pro-inflammatory cytokine of the IL-1 family. Similar to IL-β, it needs to be cleaved by caspase-1 to produce mature active IL-18 cytokine. Binding of IL-18 to its receptor will recruit MyD88 and activate the NFκB signaling pathway, leading to the production of IFN-γ, which induces Th1 immune responses [73]. IL-18 exhibits protective properties for the host in the early stages of pathogen infection. However, overproduction of IL-18 by the host or an overreaction of the host to IL-18 may lead to an excessive inflammatory response. IL-18 have been found to correlate with disease severity in sepsis, SLE, Crohn's disease and heart failure [74]. A meta-analysis have showed that IL-18 level was much higher in SLE patients compared to healthy individuals.Therefore, there may also be an association between IL-18 and autoimmune diseases [75].
IL-9	Previous studies have found that in the japonicum Schistosoma infected mice, Th9 cells and IL-9 increased rapidly at the fourth week, peaked at the seventh week and then began to decline. The levels were positively correlated with granulomatous inflammation [76]. IL-9 appeared earlier and at a significantly higher level than IL-4. It promoted the activation of hepatic stellate cells and induced the production of collagen I and III, which induced the development of liver granulomatous inflammation. Liver inflammation and fibrosis would be reduced after neutralization of IL-9 [61].
IL-17; IL-23	IL-17 is primarily a pro-inflammatory cytokine that orchestrates protection against infection by inducing antimicrobial peptide release, granuloma formation and neutrophil recruitment [33]. Higher concentrations of IL-17 were also found in schistosoma infected patients with advanced liver fibrosis [15]. IL-23 is associated with numerous autoimmune diseases and promotes autoimmune B cell responses. For example, both IL-23 and IL-17 are elevated in SLE, SS, and RA patients, and the

	IL-23/IL-17 axis correlates with the severity and chronic course of RA. The IL-23/IL-17 axis is protective against some infections such as bacteria and fungi, however，its dysregulation can lead to chronic inflammation and autoimmune responses [32-33]. Shainheit et al. Have showed by in vitro experiments that IL-23 increased and peaked after schistosome egg stimulation, followed by an increase of IL-1βand IL-17. This study suggested that differentiation of Th17 cells and IL-17 production are initiated by IL-23 and amplified by IL-1β. IL-23/IL-17 axis is one of the important mechanisms leading to severe schistosomiasis [18].
IL-21	IL-21 is mainly produced by follicular helper T cells and Th17 cells. Its expression is affected by various of factors and signaling pathways, for example, IL-6 and IL-7 promote its expression mainly through JAK/STAT, MAPK and some genes to regulate immune response [77]. IL-21 promotes antibody production by inducing the differentiation of B cells into plasma cells [66]. Wang et al. also found that IL-21 can effectively induce CD11chiT-bet+ B cells, then promote the differentiation of these cells into Ig-secreting autoreactive plasma cells. Hence, overexpression of IL-21 may promote autoimmunity [78].Blocking IL-21-related signaling pathways may have potential as a therapeutic target for autoimmune diseases [77].
IL-22	IL-22 and IL-17 have a synergistic effect in regulating the mucosal barrier by promoting antimicrobial peptide production and neutrophil recruitment. It is essential in maintaining barrier homeostasis against intestinal pathogens and commensal bacteria. However, it is worth noting that IL-17 tends to be pro-inflammatory and potentially pathogenic, whereas IL-22 exhibits mainly regenerative and protective effects and is involved in maintaining tissue homeostasis, tissue repair and wound healing [79]. IL-22 also can activate anti-apoptotic pathway and promote hepatocyte regeneration, thereby protecting the liver from hepatitis and steatosis [33]. However, the level of IL-22 show differences in different types of autoimmune diseases as well as in different stages of disease. Moreover, it have a dual role, both pathogenic and protective; therefore, the function of IL-22 depends on the type of inflammatory response, the affected tissue and the concentration and duration of IL-22 itself [80].
IL-27	IL-27 is produced by APCs in response to host and pathogen-derived inflammatory signals. It inhibits the development of pathogenic inflammatory responses by limiting the production of IL-2. It was found that during acute toxoplasmosis, the frequency of IL-2+CD4+ T cells was higher in IL-27R-deficient mice [81]. Generally, the production of IL-27 is mainly triggered by TLRs and IFN-γ. IL-27 has a dual role. It has both promoting and inhibitory effects on CD4+ T cell proliferation or recruitment, regulation of Th cell responses and Treg cell homeostasis, which are related to the time course of immune responses and disease stages, the interaction between the molecule and IL-27, differential activation of downstream signaling and different experimental models [82].
